# Maximally nonlocal Clauser-Horne-Shimony-Holt scenarios

**DOI:** 10.1038/s41598-018-24970-3

**Published:** 2018-05-08

**Authors:** Jesús Urías, José Manuel Méndez Martínez

**Affiliations:** Instituto de Física, UASLP, San Luis Potosí, SLP Mexico

## Abstract

The key feature in correlations established by multi-party quantum entangled states is nonlocality. A quantity to measure the average nonlocality, distinguishing it from shared randomness and in a direct relation with no-signaling stochastic processes (which provide an operational interpretation of quantum correlations, without involving information transmission between the parties as to sustain causality), is proposed and resolved exhaustively for the quantum correlations established by a Clauser-Horne-Shimony-Holt setup (or CHSH box). The amount of nonlocality that is available in a CHSH box is measured by its proximity to the nearest Popescu-Rohrlich set of causal stochastic processes (aka a PR box) in the no-signaling polytope, related by polyhedral duality to Bell’s correlation function. The proposed amount of average nonlocality is an entanglement monotone with a simple relation to concurrence. We provide the optimal setup vectors of a maximally nonlocal CHSH box for any entangled pair. The strongest nonlocality is the fraction $\sqrt{2}-\,1\approx 0.414$ of a PR box, attained by maximally entangled qubit pairs. The most economical causal stochastic process reproducing any maximally nonlocal CHSH box is developed. Data produced by a computer implementation of the simulator agrees with the quantum mechanical formulas.

## Introduction

The interest in quantum correlations as an information-processing resource^1–3^ puts under consideration the problem of finding the experimental setup that maximizes the strength of non-locality that is achievable from a given multi-party quantum state being shared in a Bell scenario. But first, how to size up the strength of the non-locality that might be present in quantum correlations^4^?

In a bipartite (finite ab initio) Bell scenario involving two dichotomic measurements per party (a Clauser-Horne-Shimony-Holt (CHSH) setup^5^ for short) quantum correlations may be stronger than just shared classical information, implying that an instant form of causal (non-communicating) nonlocality between the two party locations is involved. Thus, if no communication between the parties is supported, what is the physical resource that is responsible for non-locality in quantum bipartite setups? Entanglement is necessary but it is certainly not enough.

The measure of a physical setup as a resource is determined by the largest operational outcome that, in principle, the physical setup is able to provide. Traditionally, the interest was to distinguish classical from quantum correlations in Bell scenarios and it was then natural to quantify non-locality as the maximum violation of a Bell inequality allowed by a given quantum state. Nowadays, operational interests in quantum information theory might decide about the unit to quantify non-locality resources. The current views on Bell nonlocality are reviewed in^6^ and the resource-theory point of view in^4^. Currently, the PR-box is a choice^7^ to measure the amount of non-locality that is supplied by bipartite setups sharing qubit pairs.

The PR-box is an elementary (but hypothetical) building block in probability theory that combines causality and nonlocality in one unit. It was first advanced by Popescu and Rohrlich (PR)^8^ and then was proved elementary in the no-signaling formalism^6^. The PR box has become a new information-theoretic entity with the clear meaning of a non-reducible (atomic) form of causal (non-communicating) nonlocality. For instance, an unlimited number of PR boxes (together with a fixed amount of classical communication) allows two parties to give a simple solution to any communication complexity problem^9^.

Our theoretical framework for non-locality in quantum correlations in finite setups is the no-signaling formalism. This choice leaves off the discussion any form of noncausal non-locality: classical communication does not have a place in the formalism. Any *finite* causal-correlational setup—as the CHSH setup—is characterized in the no-signaling formalism by a point in the polytope of no-signaling stochastic matrices (or no-signaling boxes). The no-signaling polytope for the CHSH setup is cut by the CHSH inequalities producing the CHSH facets. The vertices the cuts leave outside are the nonlocal PR boxes. The skeleton of the CHSH polytope was computed in^10^ and the adjacency of PR boxes in the skeleton is given in Table A2 of the Appendix. Table A2 shows that every PR box is the apex of an 8-simplex with a CHSH facet as its base.

Although the non-locality produced by the PR-box per se is stronger-than-quantum, it was shown in^11^ that it can be diminished (assisted with shared randomness, without introducing mixtures) to reproduce (without communication) the quantum correlations attainable by the singlet state in an operationally free (or continuous) binary bipartite setup. In Section 5 we demonstrate that one PR box is enough too to reproduce the quantum correlations in any CHSH setup sharing qubit pairs in any partially entangled pure state.

Thus, the PR box demonstrates by itself that, as an information-theoretic operational unit, is capable to distinguish quantum from classical correlations in binary bipartite setups. Here we prove that in every CHSH setup the PR box is a physically meaningful ruler to measure the average amount of non-locality it yields.

For CHSH setups, our questioning at the beginning is rephrased in the no-signaling formalism as, how much of a PR box is realizable by a given quantum entangled qubit pair? Our way to answer this question starts in Section 2 where the quantum probabilities of coincidences in a CHSH setup—sharing a qubit pair in an arbitrary pure state—are given the form of a no-signaling stochastic matrix (that we call a Q box), which puts the CHSH setup as a point in the polytope of no-signaling boxes. Next in Section 3, we introduce a novel measure of the CHSH box *Q* as a resource of bipartite nonlocality.

For any finite causally correlated multi-party setup, we propose to measure the amount of nonlocality it yields by the proximity (accepting the usual *L*_2_ distance for probability space) of its no-signaling stochastic matrix *Q* to the nearest nonlocal vertex of the no-signaling polytope. The nonlocal box to be sized is located in the no-signaling polytope by the convex combination with the least number of extreme (elementary) boxes, including in the combination only the closest non-local vertex (just one PR box in the case of CHSH setups), the vertex that is going to fix the amount and nature of nonlocality. A similar way of weighing the content of nonlocality in Bell scenarios is proposed in^12^ as a linear program in the *L*_1_ metric, aka the classical trace distance. On the other hand, we should note that characterizing nonlocality in continuous setups is a subject that lies beyond polyhedral convex geometry^13^. Models of quantum nonlocality in unrestricted continuous setups have been considered in^11,14–17^.

For finite setups, convex geometry gives a statistical meaning and an operational interpretation to our measure of non-locality. We illustrate by example these two subjects in Sections 4 and 5. But first, in Section 3 we find that the CHSH quantum matrix *Q*—as a point in the no-signaling polytope–is located within one of the PR non-local 8-simplices. We treat the case of CHSH setups lying in the neighborhood of the prototypical PR box^8^ (with stochastic matrix denoted by *R*). Other geometries are treated similarly.

A point inside a simplex has a *unique representation* in barycentric coordinates. We expand *Q* in Section 3 as the unique convex combination of 8 local vertices and the PR box *R*. The PR coefficient in the barycentric expansion of *Q* is denoted by *N*_*Q*_ ∈ [0, 1]. Geometrically, *N*_*Q*_ is a measure of its proximity to *R* (with the *L*_2_ distance). Statistically, *N*_*Q*_ is the probability for box *Q* to behave nonlocally, as the PR box *R* does. Operationally, the convex combination of elementary (extreme) no-signaling boxes is interpreted as a set of stochastic processes. Such a full and definite physical significance is what we want of a measure of the causal nonlocality being supplied by *Q* in the average. The PR box appears thus as the information-theoretic choice for the unit of causal nonlocality. The only no-signaling box *P* with *N*_*P*_ = 1 is the PR box *R*.

Our Lemma 3.1 states that the average strength of nonlocality *N*_*Q*_ is the probability to find the CHSH quantum box *Q* behaving as the PR box does: *N*_*Q*_ is the fraction of times the nonlocal box *Q* realizes a PR box in the average. The complementary fraction of times the quantum box *Q* behaves locally. This statistical description was first discussed and termed a 2-species model by (with due respect) EPR-bis in^14^. We illustrate this point with an example in Section 5.

In Section 2 we have discovered that the Bell correlation functions are the linear functionals that place PR boxes and CHSH facets in duality^18^. For the prototypical PR box the Bell correlation linear functional on *Q* is denoted [*Q*]. Then, the two corresponding CHSH inequalities are |[*Q*]| ≤ 2, and for the PR box [*R*] = 4, which is the largest Bell correlation for a no-signaling box. A consequence of polyhedral duality we find in Section 3 is the relation between the average measure of causal nonlocality and Bell correlation: ${N}_{Q}=\frac{1}{2}[Q]-1$.

In Section 4 we find the optimal geometry (the set of setup vectors) that maximizes the average strength of bipartite non-locality that is realizable in a CHSH setup from two qubits in an arbitrary state *ψ*. The optimization results constitute our Lemma 4.1. The maximal average nonlocality ${N}_{{Q}_{\psi }}$ that is available from state *ψ*, in terms of Wootters’ concurrence *W*_*ψ*_, is given by ${N}_{{Q}_{\psi }}=\sqrt{1+{W}_{\psi }}-1\le \sqrt{2}-1\approx 0.414$, which is an increasing function of concurrence, proving that ${N}_{{Q}_{\psi }}$ is an entanglement monotone. Optimization shows us that maximally nonlocal CHSH boxes are restricted to lie in the no-signaling polytope within a 4-dimensional simplex (denoted Δ^4^) with apex the PR box *R*. We show the locus of maximally nonlocal CHSH boxes as a function of entanglement, projected into the five 3-D facets of Δ^4^.

In Section 5 the barycentric expansion of the CHSH box—supporting the measure of nonlocality ${N}_{{Q}_{\psi }}$—is interpreted as a 2-species stochastic causal simulator of the quantum correlations produced by the CHSH box. The simulator in Section 5 has the least of elementary no-signaling boxes and does not involve any form of classical communication between the parties, the PR box is the only source of nonlocality. Data produced with a computer implementation of the simulator agrees with the quantum mechanical formulas.

## The No-Signaling Polytope of CHSH Scenarios

The players in a CHSH scenario (Alice and Bob) are identified by the labels *i* = 1 and *i* = 2, respectively. They share in the setup a pair of qubits (prepared in the quantum state |*ψ*〉) and have access each to a local binary input *x*_*i*_ ∈ {0, 1} allowing them to choose one out of two setup vectors, ${\overrightarrow{a}}_{{x}_{1}}=({a}_{x}({x}_{1}),{a}_{y}({x}_{1}),{a}_{z}({x}_{1}))$ for Alice (*i* = 1) and ${\overrightarrow{b}}_{{x}_{2}}=({b}_{x}({x}_{2}),{b}_{y}({x}_{2}),{b}_{z}({x}_{2}))$ for Bob (*i* = 2), to align the quantization axes of the local observables ${\sigma }_{{\overrightarrow{a}}_{{x}_{1}}}={\overrightarrow{a}}_{{x}_{1}}\cdot \overrightarrow{\sigma \,}$ and ${\sigma }_{{\overrightarrow{b}}_{{x}_{2}}}={\overrightarrow{b}}_{{x}_{2}}\cdot \overrightarrow{\sigma \,}$. The local outcomes may result in one of the values ${(-1)}^{{w}_{i}}$, with *w*_*i*_ ∈ {0, 1}. The CHSH setup has sixteen configurations (*w*_1_*w*_2_; *x*_1_*x*_2_) with probability

1 $$P({w}_{1}{w}_{2}|{x}_{1}{x}_{2})=\langle \psi |T({w}_{1}|{x}_{1})\otimes T({w}_{2}|{x}_{2})|\psi \rangle ,$$

where the $T({w}_{i}|{x}_{i})={\textstyle \tfrac{1}{2}}(1+{(-1)}^{{w}_{i}}{\sigma }_{{\overrightarrow{v}}_{{x}_{i}}})$ are orthogonal projections.

The quantum probabilities (1) satisfy the no-signaling property (or the impossibility to use correlations to exchange information between the parties^6^)

2 $$\begin{array}{cc}P({w}_{1}|{x}_{1}) & \,=\,\sum _{{w}_{2}}P({w}_{1}{w}_{2}|{x}_{1}\,0)=\sum _{{w}_{2}}P({w}_{1}{w}_{2}|{x}_{1}1),\\ P({w}_{2}|{x}_{2}) & \,=\,\sum _{{w}_{2}}P({w}_{1}{w}_{2}|0\,{x}_{2})=\sum _{{w}_{2}}P({w}_{1}{w}_{2}|1{x}_{2}).\end{array}$$

Then, Alice’s marginal probabilities are independent of Bob’s input choice and conversely. Besides, $\sum _{{w}_{1}{w}_{2}}P({w}_{1}{w}_{2}|{x}_{1}{x}_{2})=1$ and *P*(*w*_1_*w*_2_|*x*_1_*x*_2_) ≥ 0.

The foregoing equations (no-signaling and normalization) reduce the sixteen probabilities to eight independent variables and the inequalities (the non-negativity conditions) define half-spaces in 16-Euclidean space. The region in 16-space where all conditions are satisfied is the 8-dimensional polytope of the 4 × 4 no-signaling stochastic matrices (or boxes), with entries *P*(*w*_1_*w*_2_|*x*_1_*x*_2_): the binary word *x* = *x*_1_*x*_2_ is the matrix row index and the binary word *w* = *w*_1_*w*_2_ the column index. The probabilities in (1) are the entries of the quantum box *Q* supplied by state *ψ* in the CHSH setup.

The stochastic matrices that are the vertices of the CHSH no-signaling polytope were computed with the enumeration algorithm^10^. The matrix entries *P*(*w*_1_*w*_2_|*x*_1_*x*_2_) of the vertex matrices are presented in Table A1 of the Appendix. All the local vertices in Table A1 (that we call *B*-boxes) are parameterized in the single formula

3 $${B}_{\alpha a\beta b}\,=(\begin{array}{cc}a+1 & a\\ \alpha +a+1 & \alpha +a\end{array})\otimes (\begin{array}{cc}b+1 & b\\ \beta +b+1 & \beta +b\end{array})\,({\rm{m}}{\rm{o}}{\rm{d}}\,2),$$

with *αaβb* ∈ {0, 1}^4^. *B*-boxes factor out into local 2 × 2 deterministic transition matrices. Any mixture of product matrices is a local box.

The eight vertices of the no-signaling polytope in Table A1 that are stochastic matrices that cannot be factorized are the (nonlocal) PR boxes. We denote them by *R*_*ijk*_, with *ijk* ∈ {0, 1}^3^, and have entries parameterized in the following form,

4 $${R}_{ijk}({w}_{1}\,{w}_{2}|{x}_{1}\,{x}_{2})\,=\,\{\begin{array}{cc}1/2, & {w}_{1}+{w}_{2}=({x}_{1}+i)({x}_{2}+j)+k\,\,({\rm{m}}{\rm{o}}{\rm{d}}\,2)\\ 0, & {\rm{o}}{\rm{t}}{\rm{h}}{\rm{e}}{\rm{r}}{\rm{w}}{\rm{i}}{\rm{s}}{\rm{e}}\end{array}\,.$$

Every PR box in the polytope connects through edges with eight of the B-boxes, conforming an 8-simplex. The adjacency of PR boxes in the polytope is given in Table A2. The prototypical PR box *R*_000_ and its eight neighbors are highlighted with an asterisk in Table A1. We denote by ${ {\mathcal B} }_{\ast }$ the set of *B*-boxes with an asterisk in Table A1.

Every pair of antipodal PR boxes defines in the no-signaling polytope the axis *σ*_*ij*_ = 2(*R*_*ij*0_−*R*_*ij*1_) of nonlocality. Each axis (a pair of PR boxes) is dual to a pair of CHSH facets through the inner product (*σ*_*ij*_, *Q*), where (*A*, *B*) = tr(*A*^*T*^*B*) for real matrices. This fact is confirmed using the matrices *R*_*ijk*_ in (4) and the definition 〈*x*_1_*x*_2_〉 = *P*(00|*x*_1_*x*_2_) + *P*(11|*x*_1_*x*_2_) − *P*(01|*x*_1_*x*_2_) − *P*(10|*x*_1_*x*_2_). Then, for every binary word *ij* there is the CHSH inequality |(*σ*_*ij*_, *Q*)| ≤ 2 for any local no-signaling box *Q*,

The prototypical axis of nonlocality of our interest is *σ*_00_ (or just *σ*), for which the scalar product

5 $$[Q]:\,\,=(\sigma ,Q)=\langle 00\rangle +\langle 01\rangle +\langle 10\rangle -\langle 11\rangle ,$$

is the usual Bell correlation [*Q*] of box *Q*. Notice that the Bell correlation [⋅] is just the linear functional that puts the corresponding CHSH facets in duality with the axis of nonlocality *σ*. The PR box *R*_000_ (or just *R*) has [*R*] = 4, the largest Bell correlation for a no-signaling box in the polytope. Since [*B*] = 2 for every box $B\,\in \,{ {\mathcal B} }_{\ast }$ and $|{ {\mathcal B} }_{\ast }|=8$, then the affine span of ${ {\mathcal B} }_{\ast }$ is the hyperplane in 8-space that supports the CHSH facet that is the dual of *R*.

## The Average Causal Nonlocality of a CHSH Quantum Box

We’ll look for the maximally nonlocal CHSH setup for the very general quantum state

6 $$|\psi \rangle ={e}^{i{\alpha }_{1}}\,\cos \beta \,|01\rangle +{e}^{i{\alpha }_{2}}\,\sin \beta \,|10\rangle \,,\,\beta \,\in \,[0,\,\pi /2].$$

The content of entanglement in |*ψ*〉 is measured by its concurrence ${W}_{\psi }={\sin }^{2}2\beta$.

The probability entries of the quantum box *Q* that is supplied by the CHSH setup with the general state *ψ* in (6), are calculated with (1), resulting in

7 $$\begin{array}{cc}P({w}_{1}{w}_{2}|{x}_{1}{x}_{2})\,=\, & \frac{1}{4}(1-{(-1)}^{{w}_{1}+{w}_{2}}{a}_{z}({x}_{1}){b}_{z}({x}_{2})\\  & +\,({(-1)}^{{w}_{1}}{a}_{z}({x}_{1})-{(-1)}^{{w}_{2}}{b}_{z}({x}_{2}))\,\cos 2\beta \\  & +\,{(-1)}^{{w}_{1}+{w}_{2}}(({a}_{x}({x}_{1}){b}_{x}({x}_{2})+{a}_{y}({x}_{1}){b}_{y}({x}_{2}))\,\cos \alpha \\  & -({a}_{x}({x}_{1}){b}_{y}({x}_{2})-{a}_{y}({x}_{1}){b}_{x}({x}_{2}))\,\sin \alpha )\,\sin 2\beta ),\end{array}$$

where *α* = *α*_2_ − *α*_1_.

For no-signaling boxes in the vicinity of the PR box *R* it is convenient to choose vertex *R* as the origin and adopt the set of edges ${\mathcal B} =\{\beta =B-R:B\,\in \,{ {\mathcal B} }_{\ast }\}$ (see the adjacency Table A2 in the Appendix) as a vector basis. Let ${ {\mathcal B} }^{r}$ be the reciprocal to basis ${\mathcal B}$. By convexity we have that cone $( {\mathcal B} )$ covers the polytope. Thus matrix *Q* relative to *R* is expanded in the ${\mathcal B}$ basis as $Q-R={\sum }_{\beta }\beta {C}_{\beta }$, with coefficients *C*_*β*_ = (*β*^*r*^, *Q* − *R*) ≥ 0. The expansion provides the barycentric representation

8 $$\begin{array}{cc}Q & \,=\sum _{B\,\in \,{{\mathscr{B}}}_{\ast }}B{C}_{\beta }\,+\,(1-\sum _{\beta }{C}_{\beta })\,R\\  & =\sum _{B\,\in \,{{\mathscr{B}}}_{\ast }}B{C}_{\beta }\,+\,(\frac{1}{2}[Q]-1)\,R\,,\end{array}$$

where we have used the Bell correlations [*B*] = 2 and [*R*] = 4 to get that all coefficients add up to one,

9 $$\sum _{\beta }{C}_{\beta }+(\frac{1}{2}[Q]-1)=1.$$

Recall that the Bell correlation [⋅] used in (8) and in (9) is the linear functional that puts the PR box and the CHSH facet in duality.

By definition, the expansion (8) of any no-signaling box has *C*_*β*_ ≥ 0 for all $\beta \,\in \, {\mathcal B}$, while the PR coefficient ${N}_{Q}:=\frac{1}{2}[Q]-1$ is positive only when box *Q* is in the interior of the 8-simplex with *R* as its apex, since then [*Q*] > 2. Our definition of the average strength of causal nonlocality of box *Q* is the coefficient of the PR box *R* in the barycentric representation (8),

10 $${N}_{Q}={\textstyle \tfrac{1}{2}}[Q]-1,\,{\rm{f}}{\rm{o}}{\rm{r}}\,[Q] > 2\,,$$

and *N*_*Q*_ = 0 otherwise. The connection of the average strength of causal nonlocality with the Bell correlation of box *Q* established in (10) is a consequence of polyhedral duality. It does not suffer of any ambiguity given the uniqueness of the barycentric expansion (8). Besides, only the PR box has the largest amount of nonlocality, *N*_*R*_ = 1. The results are summarized in the following.

### Lemma 3.1.

*The no-signaling box Q that is provided by a CHSH setup with the quantum state* (6) *is the mixture*

11 $$Q=(1-{N}_{Q})L+{N}_{Q}R$$

*of a local box L and the PR box R. The average strength of causal nonlocality of box Q is* ${N}_{Q}=\frac{1}{2}[Q]-1$, *of box L is N*_*L*_ = 0 *and N*_*R*_ = 1.

### Proof

: Normalize the *C*_*β*_ coefficients in (8) to ${c}_{\beta }={C}_{\beta }/\sum {C}_{\beta }$. Then, using $\sum {C}_{\beta }=2-\,\frac{1}{2}[Q]$ from (9), we obtain from (8) that $Q=(2-{\textstyle \tfrac{1}{2}}[Q])\sum {c}_{\beta }B+({\textstyle \tfrac{1}{2}}[Q]-1)\,R$, where $L=\sum {c}_{\beta }B$ and $\sum {c}_{\beta }=1$. That *N*_*L*_ = 0 follows from $[L]=\sum {c}_{\beta }[B]=2$ and definition (10).□

Following the procedure leading to (8) on our quantum no-signaling box *Q*, with probability entries in (7), we get the convex combination

12 $$\begin{array}{cc}Q & \,=\,\frac{1}{4}((1+\langle 11\rangle +\,\cos 2\beta \,({a}_{z}(1)-{b}_{z}(1)))\,{B}_{0000}+(1+\langle 11\rangle -\,\cos 2\beta \,({a}_{z}(1)-{b}_{z}(1)))\,{B}_{0101}\\  & +\,(1-\langle 01\rangle +\,\cos 2\beta \,({a}_{z}(0)+{b}_{z}(1)))\,{B}_{0010}+(1-\langle 01\rangle -\,\cos 2\beta \,({a}_{z}(0)+{b}_{z}(1)))\,{B}_{0111}\\  & +\,(1-\langle 10\rangle -\,\cos 2\beta \,({a}_{z}(1)+{b}_{z}(0)))\,{B}_{1000}+(1-\langle 10\rangle +\,\cos 2\beta \,({a}_{z}(1)+{b}_{z}(0)))\,{B}_{1101}\\  & +\,(1-\langle 00\rangle +\,\cos 2\beta \,({a}_{z}(0)+{b}_{z}(0)))\,{B}_{1011}+(1-\langle 00\rangle -\,\cos 2\beta \,({a}_{z}(0)+{b}_{z}(0)))\,{B}_{1110})\\  & +\,{N}_{Q}R\,,\end{array}$$

where the coefficients *C*_*β*_ are easily identified and the stochastic matrices *B*_*αaβb*_ involved in (12) (elements of ${ {\mathcal B} }_{\ast }$) are highlighted with an asterisk in Table A1 (see the parameterized form in (3) too). The pair correlations in the CHSH setup are

13 $$\begin{array}{cc}\langle {x}_{1}{x}_{2}\rangle \,=\, & -{a}_{z}({x}_{1}){b}_{z}({x}_{2})+\,\sin 2\beta \,(({a}_{x}({x}_{1}){b}_{x}({x}_{2})+{a}_{y}({x}_{1}){b}_{y}({x}_{2}))\,\cos \alpha \\  & -({a}_{x}({x}_{1}){b}_{y}({x}_{2})-{a}_{y}({x}_{1}){b}_{x}({x}_{2}))\,\sin \alpha ).\end{array}$$

The average strength of causal nonlocality, *N*_*Q*_, of the CHSH box *Q* provided by state *ψ* in (6), is to be computed by substituting the pair correlations (13) in the Bell correlation (5) and then using definition (10). The result for *N*_*Q*_ is an involved function of the setup vectors and the angle of entanglement *β*.

## Maximally Nonlocal CHSH Setups

The stochastic matrix *Q* of the correlational setup that is produced when the quantum state *ψ* in (6)—containing an arbitrary amount of entanglement—is being shared in a CHSH setup is given in (12). The average measure of causal nonlocality of the quantum box *Q*, *N*_*Q*_ introduced in Lemma 3.1, is computed using (10), (5) and (13). Thus, we are in position to tackle the problem of finding the optimal setup vectors ${\overrightarrow{a}}_{opt}({x}_{1})$ and ${\overrightarrow{b}}_{opt}({x}_{2})$ that maximize the average strength of non-locality that is realizable by state *ψ*.

The optimal setup vectors and the corresponding measure of nonlocality and optimal local box *L* (denoted $\overline{L}$ when optimal) are given in the following.

### Lemma 4.1.

*The CHSH quantum no signaling box Q in* (12) *showing the maximum strength of nonlocality is*

14 $${Q}_{\psi }=(2-{V}_{\psi })\bar{L}+({V}_{\psi }-1)R,$$

*where* ${V}_{\psi }:\,\,=\sqrt{1+{W}_{\psi }}$ *is the Bell violation factor;* ${N}_{{Q}_{\psi }}={V}_{\psi }-1$ *is the maximal strength of nonlocality for ψ; and optimal box* $\bar{L}$ *is the local box L in Lemma 3.1, evaluated at the optimal setup vectors*

15 $$\begin{array}{cc}{\overrightarrow{a}}_{opt}(0) & \,=\,\hat{z\,}\,,\,{\overrightarrow{a}}_{opt}(1)=(\cos \,\theta )\,\hat{x\,}+(\sin \,\theta )\,\hat{y\,}\,,\\ {\overrightarrow{b}}_{opt}({x}_{2}) & \,=\,\frac{1}{{V}_{\psi }}((-1{)}^{{x}_{2}}\,(\sin \,2\beta )\,\hat{r\,}-\,\hat{z\,}\,)\,,\end{array}$$

where $\hat{r\,}:\,\,=\,\cos \,(\theta -\alpha )\,\hat{x\,}+\,\sin \,(\theta -\alpha )\hat{y\,}$ is a unit vector and *θ* ∈ [0, 2*π*] is any angle.

### Proof

: Maximization of the average causal nonlocality *N*_*Q*_ in (10) is equivalent to maximization of the Bell correlation [*Q*]. The Bell correlation [*Q*] as a function of the setup vectors is obtained by replacing the pair correlations (13) in (5). We require the maximum value of [*Q*] for setup vectors subjected to lie on the unit sphere. The method of Lagrange multipliers yields the optimal unit ${\overrightarrow{a}}_{opt}(0)=(0,\,0,\,{a}_{z})$ with ${a}_{z}=\pm 1$, ${\overrightarrow{a}}_{opt}(1)\,=\,(\cos \,\theta ,\,\sin \,\theta ,\,0)$ with $\theta \in \mathrm{[0,}\,2\pi ]$ an arbitrary angle, and ${\overrightarrow{b}}_{opt}({x}_{2})=\lambda \,((-1{)}^{{x}_{2}}\,\sin \,2\beta \,\overrightarrow{r\,}-{a}_{z}\hat{z\,})(1+{\sin }^{2}2\beta {)}^{-1/2}$, with *λ* = ±1. Putting the optimal vectors back into [*Q*] yields the extreme values $[Q]=2\lambda \sqrt{1+{\sin }^{2}2\beta }$. For the maximum value of [*Q*] let *λ* = 1. We are free to choose *a*_*z*_ = 1.□

The stochastic matrix *Q*_*ψ*_ in Lemma 4.1 is the maximally nonlocal CHSH box for the given state *ψ*. The result and main interest in Lemma 4.1 is not the upper bound but the location of the optimal box in the no-signaling polytope, equation (14). The upper bound is well known. It has been obtained in the classic reference^19^ and by the spectral decomposition of Bell operators^20,21^. An extra bonus of Lemma 4.1 is the geometry of the optimal CHSH setup for *ψ*.

**Figure 1:**
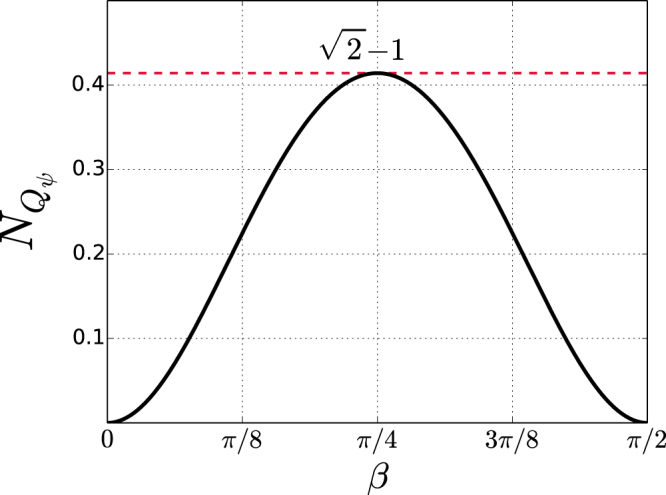
Maximal nonlocality ${N}_{{Q}_{\psi }}$ attainable in a CHSH setup by a state *ψ* with angle of entanglement *β*. The quantum limit $\sqrt{2}-1\approx 0.414$ is attained by a state with concurrence *W*_*ψ*_ = 1.

The nonlocality ${N}_{{Q}_{\psi }}$ attainable in the optimal CHSH setup for state *ψ* as a function of the angle of entanglement *β* is shown in Fig. 1. The corresponding Bell correlation as given by (14) is [*Q*_*ψ*_] = 2*V*_*ψ*_, that proves ${V}_{\psi }=\sqrt{1+{W}_{\psi }}\le \sqrt{2}$ is the Bell violation factor. The maximal average strength of causal nonlocality ${N}_{{Q}_{\psi }}=\sqrt{2}-1\approx 0.4142$ is attained by a maximally entangled state *ψ* with concurrence *W*_*ψ*_ = 1 when shared in its optimal CHSH setup (15).

The local box $\bar{L}$ in the mixture (14) that reproduces the maximally nonlocal *Q*_*ψ*_, is obtained by substituting the optimal setup vectors (15) in the local part of the expansion (12) of *Q*. The result is the convex combination

16 $$\bar{L}=\sum _{{s}_{1}{s}_{2}}{p}_{{s}_{1}{s}_{2}}{b}_{{s}_{1}{s}_{2}},$$

which is a local box lying inside a 3-simplex with vertices

17 $${b}_{00}={\textstyle \tfrac{1}{2}}({B}_{0000}+{B}_{1000})=(\begin{array}{cc}1 & 0\\ 1/2 & 1/2\end{array})\otimes (\begin{array}{cc}1 & 0\\ 1 & 0\end{array})\,,$$

18 $${b}_{01}={\textstyle \tfrac{1}{2}}({B}_{0101}+{B}_{1101})=(\begin{array}{cc}0 & 1\\ 1/2 & 1/2\end{array})\otimes (\begin{array}{cc}0 & 1\\ 0 & 1\end{array})\,,$$

19 $${b}_{10}={\textstyle \tfrac{1}{2}}({B}_{0010}+{B}_{1011})={\textstyle \tfrac{1}{2}}(\begin{array}{cc}1 & 0\\ 1 & 0\end{array})\otimes (\begin{array}{cc}1 & 0\\ 0 & 1\end{array})\,+\,{\textstyle \tfrac{1}{2}}(\begin{array}{cc}1 & 0\\ 0 & 1\end{array})\otimes (\begin{array}{cc}0 & 1\\ 1 & 0\end{array})\,,$$

20 $${b}_{11}={\textstyle \tfrac{1}{2}}({B}_{0111}+{B}_{1110})={\textstyle \tfrac{1}{2}}(\begin{array}{cc}0 & 1\\ 0 & 1\end{array})\otimes (\begin{array}{cc}0 & 1\\ 1 & 0\end{array})\,+\,{\textstyle \tfrac{1}{2}}(\begin{array}{cc}0 & 1\\ 1 & 0\end{array})\otimes (\begin{array}{cc}1 & 0\\ 0 & 1\end{array})\,.$$

Written in factorized form makes evident the local character of the *b*-boxes. The Bell correlation of the *b*-boxes is $[{b}_{{s}_{1}{s}_{2}}]=2$, which is the largest value for a local box.

The mixing coefficients ${p}_{{s}_{1}{s}_{2}}$ in (16) are obtained by substituting the optimal setup vectors (15) in the *c*_*β*_ coefficients of the expansion (12) of *Q*,

21 $${p}_{{s}_{1}{s}_{2}}=\frac{1}{2{V}_{\psi }\,(2-{V}_{\psi })}\,\{\begin{array}{cc}{V}_{\psi }-{\sin }^{2}\,2\beta +{(-1)}^{{s}_{2}}\,\cos \,2\beta \,, & {\rm{i}}{\rm{f}}\,{s}_{1}=0\\ ({V}_{\psi }-1)\,(1+{(-1)}^{{s}_{2}}\,\cos \,2\beta ), & {\rm{i}}{\rm{f}}\,{s}_{1}=1\end{array}$$

and ${\sum }_{{s}_{1}{s}_{2}}{p}_{{s}_{1}{s}_{2}}=1$. Thus, $[\bar{L}]=2$ and ${N}_{\bar{L}}=0$.

The maximally nonlocal boxes *Q*_*ψ*_ are a mixture of the PR box *R* and a local box $\bar{L}$ (the mixture of local boxes in (16)). As a function of the angle of entanglement *β* of state *ψ*, the map $\beta \mapsto {Q}_{\psi }$ generates the locus of maximally nonlocal boxes in the space of stochastic matrices.

**Figure 2:**
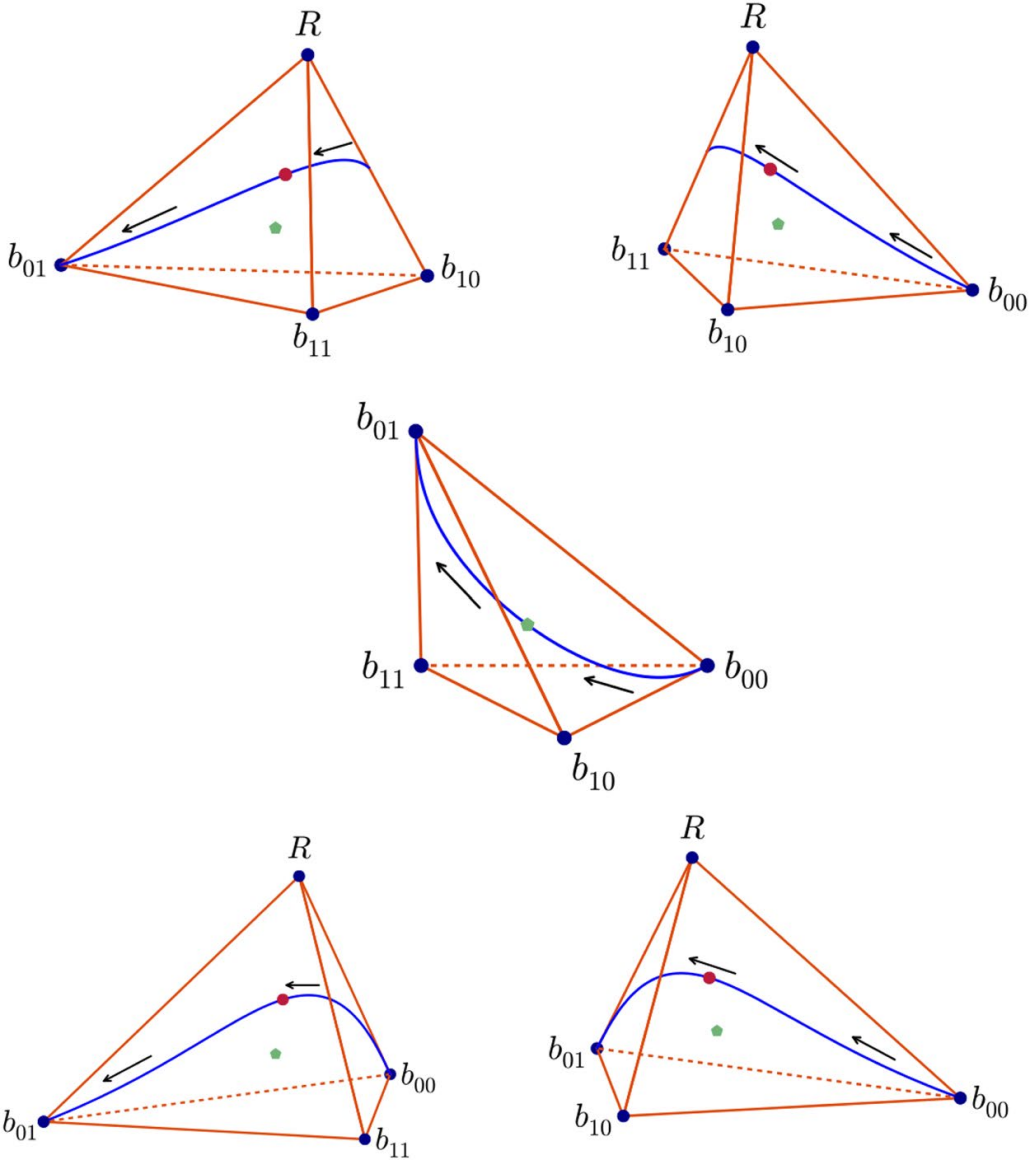
Locus of maximally nonlocal quantum boxes *Q*_*ψ*_ (in blue) projected into the five facets of the simplex Δ^4^. The box *Q*_*ψ*_ with the strongest nonlocality ${N}_{{Q}_{\psi }}$ (≈0.414, stemming from a maximally entangled state of concurrence *W*_*ψ*_ = 1 is shown as a red dot. Barycenters of the facets are shown in green.

The locus of maximally nonlocal boxes lies within the 4-simplex with apex the PR box R and base the 3-simplex with vertices the local boxes ${b}_{{s}_{1}{s}_{2}}$ in (17)–(20) (let us denote it Δ^4^). The projections of the locus of *Q*_*ψ*_ boxes onto each of the five facets (3-simplices) of Δ^4^ are shown as the blue curves in Fig. 2. The arrows along the locus indicate the direction of increasing *β*. The end points of the locus of *Q*_*ψ*_ boxes are the product boxes *b*_00_ (when *β* = 0, *V*_*ψ*_ = 1; see (21)) and *b*_01_ (when *β* = *π*/2, *V*_*ψ*_ = 1; see (21)), with stochastic matrices in (17) and (18). The box with the strongest causal nonlocality ($\sqrt{2}-1$) is marked with a red dot, which is reached when *β* = *π*/4. The green dot is the barycenter of the facet. All boxes *Q* in the 3-simplex with vertices {*b*_*ij*_} are local, with the biggest Bell correlation [*Q*] = 2 for a local box. In this facet of local boxes the barycenter and the projection of the box with the strongest nonlocality coincide.

## Maximally Nonlocal Boxes as Stochastic Processes

The no-signaling boxes and the associated average measure of causal nonlocality (10) have, by definition, a direct interpretation as a set of stochastic processes that simulate quantum correlations without using classical forms of communication between the parties.

The random variable of a box-process is the binary word *w*_1_*w*_2_ ∈ {0, 1}^2^, and every row of the associated stochastic matrix–selected with the binary word *x*_1_*x*_2_–determines a mode of operation of the box, having its own distribution of probability over the possible random outcomes for word *w*_1_*w*_2_.

For instance, depending on the choice of the product *x*_1_ ⋅ *x*_2_, the PR box *R* has two modes of operation (see the *R*_*ijk*_ matrices in Table A1 or the parameterized form in (4)). A perfectly anti-correlated mode for *x*_1_ ⋅ *x*_2_ = 1 and a perfectly correlated mode for *x*_1_ ⋅ *x*_2_ = 0. The nonlocality of the PR box relies on the switching between those two correlational modes, without allowing the parties to communicate.

The maximally nonlocal boxes *Q*_*ψ*_ are a mixture of the PR box *R* and the four ${b}_{{s}_{1}{s}_{2}}$ boxes in (17)–(20), combined in the mixture (16) as box $\bar{L}$. As 4 × 4 stochastic matrices the ${b}_{{s}_{1}{s}_{2}}$ boxes are the following,

22 $$\begin{array}{cccccc}{b}_{00} & = & (\begin{array}{cccc}1 & 0 & 0 & 0\\ 1 & 0 & 0 & 0\\ 1/2 & 0 & 1/2 & 0\\ 1/2 & 0 & 1/2 & 0\end{array})\,; & {b}_{01} & = & (\begin{array}{cccc}0 & 0 & 0 & 1\\ 0 & 0 & 0 & 1\\ 0 & 1/2 & 0 & 1/2\\ 0 & 1/2 & 0 & 1/2\end{array})\,;\\ {b}_{10} & = & (\begin{array}{cccc}1/2 & 1/2 & 0 & 0\\ 1/2 & 1/2 & 0 & 0\\ 1/2 & 0 & 0 & 1/2\\ 0 & 1/2 & 1/2 & 0\end{array})\,; & {b}_{11} & = & (\begin{array}{cccc}0 & 0 & 1/2 & 1/2\\ 0 & 0 & 1/2 & 1/2\\ 1/2 & 0 & 0 & 1/2\\ 0 & 1/2 & 1/2 & 0\end{array})\,.\end{array}$$

Every row in a *b*-matrix has at most two options with 1/2 probability each. Then, a single random bit *r* shared by the parties (with 1/2 probabilities for 0 and 1) is sufficient to reproduce the statistics of the output words of the ${b}_{{s}_{1}{s}_{2}}$ no-signaling boxes in (22) by some stochastic processes. Let us denote the output word of the ${b}_{{s}_{1}{s}_{2}}$ boxes by *v*_1_*v*_2_. The processes

23 $$\begin{array}{cc}{v}_{1} & \,=\,r{x}_{1}+{s}_{2}\,\,({\rm{m}}{\rm{o}}{\rm{d}}\,2)\,,\\ {v}_{2} & \,=\,{s}_{1}({x}_{2}+r)+{s}_{2}\,\,({\rm{m}}{\rm{o}}{\rm{d}}\,2)\,,\end{array}$$

asymptotically reproduce the probability entries in every ${b}_{{s}_{1}{s}_{2}}$ matrix. This is proved by comparison of the probabilities ${P}_{{s}_{1}{s}_{2}}({v}_{1}{v}_{2}|{x}_{1}{x}_{2})$ that follow from (23) with the entries of the *b*-matrices in (22). When *x*_1_ = 0 or *s*_1_ = 0, *v*_1_ and *v*_2_ in (23) are independent variables and ${P}_{{s}_{1}{s}_{2}}({v}_{1}{v}_{2}|{x}_{1}{x}_{2})={P}_{{s}_{1}{s}_{2}}({v}_{1}|{x}_{1}){P}_{{s}_{1}{s}_{2}}({v}_{2}|{x}_{2})$, with

$$\begin{array}{ccc}{P}_{{s}_{1}{s}_{2}}({v}_{1}=1|{x}_{1}) & \,= & \langle {v}_{1}\rangle ={\textstyle \tfrac{1}{2}}{x}_{1}+{s}_{2}(1-{x}_{1})\,,\\ {P}_{{s}_{1}{s}_{2}}({v}_{2}=1|{x}_{2}) & \,= & \langle {v}_{2}\rangle ={\textstyle \tfrac{1}{2}}{s}_{1}(1-2{s}_{2})+{s}_{2}\,,\end{array}$$

where 〈*r*〉 = 1/2. When *x*_1_ = *s*_1_ = 1, then

$${P}_{{s}_{1}=1,{s}_{2}}({v}_{1}{v}_{2}|{x}_{1}=1,{x}_{2})=\{\begin{array}{cc}1/2\,, & {v}_{2}={v}_{1}+{x}_{2}\,\,({\rm{m}}{\rm{o}}{\rm{d}}\,2)\\ 0\,, & {\rm{o}}{\rm{t}}{\rm{h}}{\rm{e}}{\rm{r}}{\rm{w}}{\rm{i}}{\rm{s}}{\rm{e}}\end{array}\,.$$

The comparison has resulted in coincidence in every case.

The optimal local box $\bar{L}$ in (16) is the mixture of the four ${b}_{{s}_{1}{s}_{2}}$ boxes, with probabilities ${p}_{{s}_{1}{s}_{2}}$ given in (21): the random bits *s*_1_ and *s*_2_ are not independent. Then, to reproduce the local box $\overline{L}$ by the mixture of processes in (23), with probabilities ${p}_{{s}_{1}{s}_{2}}$, the binary word *s*_1_*s*_2_ is in the stochastic process a piece of random information that is shared by the parties too.

**Figure 3:**
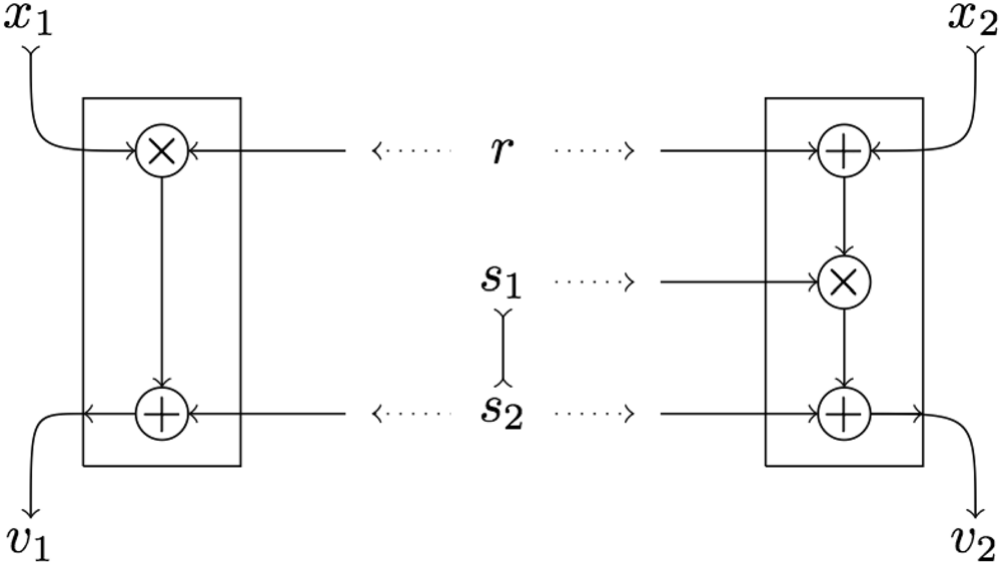
Stochastic process corresponding to the local box $\overline{L}$, Eqs (16–20). The line connecting *s*_1_ and *s*_2_ is used to indicate they are not independently random, they are tight by (21).

The process reproducing the local box $\overline{L}$ (16) is represented graphically in Fig. 3. The “input-output flow” in the diagram goes from top to bottom and the parties are far apart horizontally. The classical information that is shared by the parties consists of the three random bits *r*, *s*_1_ and *s*_2_. The two bits in the word *s*_1_*s*_2_ are not independently random.

**Figure 4:**
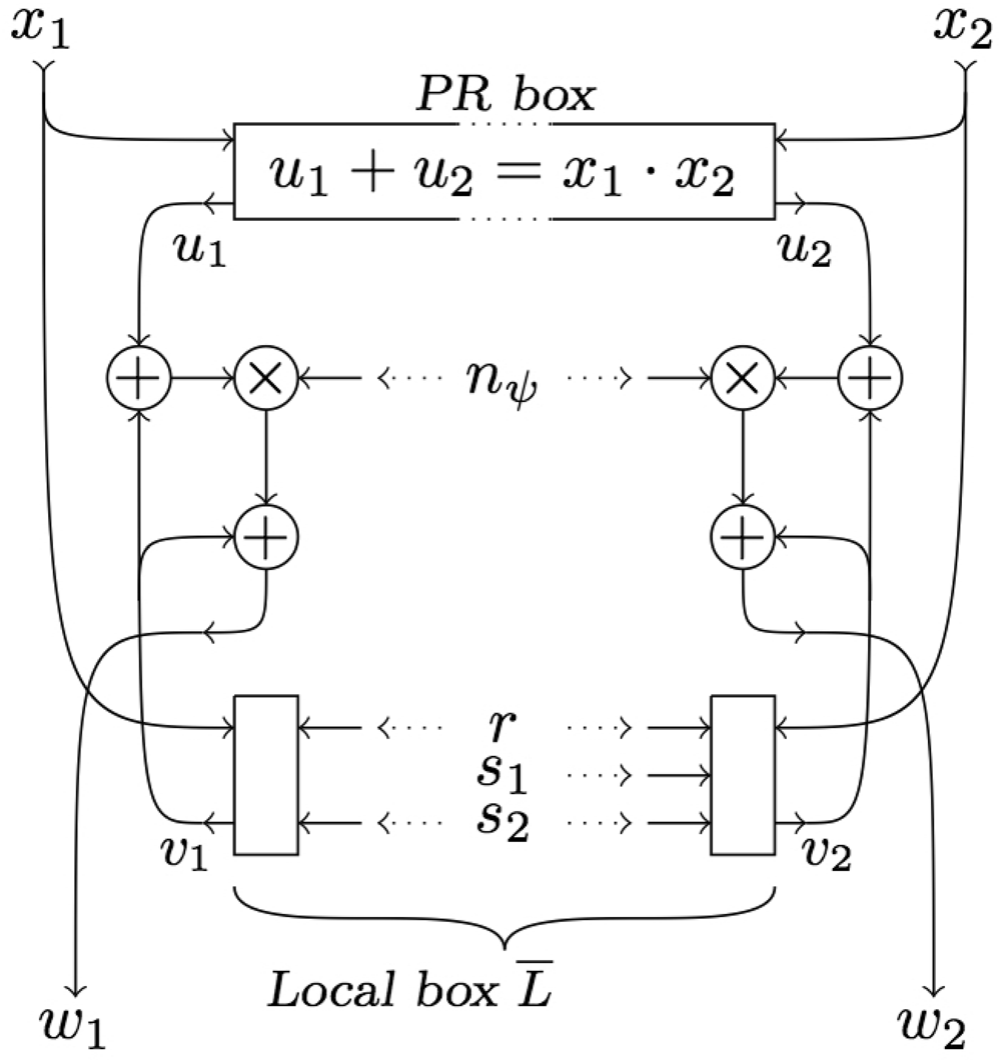
Stochastic process that reproduces, asymptotically, the mixture (14) for the maximally nonlocal quantum box *Q*_*ψ*_ in Lemma 4.1. The random bit *n*_*ψ*_ gets the value 1 with probability given by the strength of causal nonlocality ${N}_{{Q}_{\psi }}=\sqrt{1+{W}_{\psi }}-1$.

The stochastic process that simulates the maximally nonlocal quantum box *Q*_*ψ*_ derives from the mixture (14), of the local box $\overline{L}$ and the PR box *R*, with mixing probability the strength of nonlocality ${N}_{{Q}_{\psi }}$. Then, sharing a single random bit *n*_*ψ*_ is sufficient to produce the mixture of the process reproducing box $\overline{L}$ in Fig. (3) with the PR box *R*. The combined process is shown in Fig. 4.

The process in Fig. 4 reproduces the statistics of the outcomes for the binary word *w*_1_*w*_2_ of the maximally nonlocal quantum box *Q*_*ψ*_ when the probability that the random bit *n*_*ψ*_ gets the value 1, equals the average strength of causal nonlocality ${N}_{{Q}_{\psi }}={V}_{\psi }-1\le \sqrt{2}-1\approx 0.414$.

We have tested the process in Fig. 4 by comparing the pair correlations 〈*x*_1_*x*_2_〉 resulting from a computer implementation of Fig. 4 with the corresponding quantum mechanical formulas, which are obtained by substituting the optimal setup vectors (15) in the pair correlations (13). The result consists of the following quantum mechanical formulas,

24 $$\begin{array}{cc}\langle 0\,{x}_{2}\rangle  & \,=\,\frac{1}{{N}_{{Q}_{\psi }}+1}=\frac{1}{{V}_{\psi }}\,,\\ \langle 1\,{x}_{2}\rangle  & \,=\,{(-1)}^{{x}_{2}}\,{N}_{{Q}_{\psi }}\,\frac{{N}_{{Q}_{\psi }}+2}{{N}_{{Q}_{\psi }}+1}={(-1)}^{{x}_{2}}\,\frac{{V}_{\psi }^{2}-1}{{V}_{\psi }}.\end{array}$$

**Figure 5:**
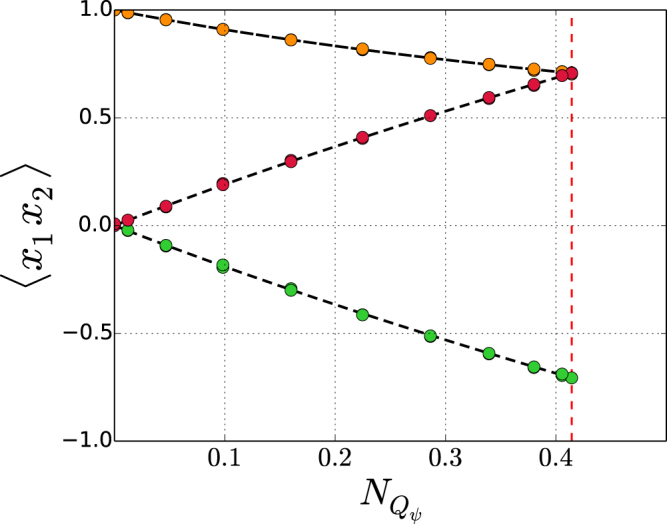
Pair correlations 〈*x*_1_*x*_2_〉 versus nonlocality ${N}_{{Q}_{\psi }}$ in the maximally nonlocal CHSH setup. Dots are data produced by a computer implementation of the stochastic process in Fig. 4: 〈00〉 = 〈01〉 in orange, 〈10〉 in red and 〈11〉 in green. The lines are the graphs of the quantum-mechanical formulas (24).

The pair correlations 〈*x*_1_*x*_2_〉 estimated numerically by the computer version of the process in Fig. 4 were compared with the formulas in (24). The comparison in Fig. 5 shows a very good agreement between the numerical results produced by the computer version of the stochastic process and the results of quantum mechanics.

## Concluding remarks

The harmonious blend of causality and nonlocality in quantum correlations is an intriguing phenomenon. Entanglement simulation was intended to quantify the nonlocality of EPR pairs in terms of the amount of communication necessary to simulate the correlations produced in a CHSH setup. However, the approach disregards causality and is not appropriate to physically understand the phenomenon. Instead, the no-signaling formalism has introduced the methods and the elements (e.g., the PR box as a basic element that is causally nonlocal) to gain understanding about violations of CHSH inequalities, without recurring to any form of communication.

In search of how and when nonlocality operates, the model for the “continuous Bohm setup” by Cerf *et al*. in^11^ behaves nonlocally all the times through the PR box. In the model^11^ shared randomness disguises nonlocality and does not dilute it in a mixture of “local and non local qubits”. That it must be so for the singlet state in a continuous setup was proved by EPR-bis in^14^.

On the opposite sense, the no-signaling formalism proves that every *finite* causally correlated setup admits a 2-species simulator^14^ that sometimes behaves locally and some others nonlocally. For “continuous” setups (those taking into account the whole set of possible measurements) the situation is not that final. The 2-species problem in continuous setups has been considered in^14–17^.

We have developed in full detail the 2-species description of correlations in CHSH setups. We have shown how convex geometry (*i*) decomposes quantum correlations into local and nonlocal parts, disclosing the nonlocal content, and (*ii*) provides the local and nonlocal resources (e.g., the PR box) to build the most economical 2-species simulators of quantum correlations, i.e., the simulators using the least number of elementary resources to compose the 2-species mixture. Figure 4 is the most economic 2-species simulator for a pair of qubits in a partially entangled state when shared in its optimal CHSH geometry.

The average measure of the amount of causal nonlocality in the class of 2-species models is the fraction of nonlocal species in the ensemble^14^. The average nonlocality provided by the simulator in Fig. 4 is ${N}_{{Q}_{\psi }}=\sqrt{1+{W}_{\psi }}-1={V}_{\psi }-1$, where *W*_*ψ*_ is *ψ*’s concurrence and *V*_*ψ*_ is the Bell violation factor. The quantum limit on the nonlocal content is ${N}_{{Q}_{\psi }}\le \sqrt{2}-1$.

The methods of convex geometry we have applied for the CHSH setups have a general value. They equally apply to decompose quantum correlations into local and nonlocal parts and to identify the necessary elementary resources for mixed states in any *finite* multi-party scenario. A hard task we foresee is the construction of the skeleton of the degenerate no-signaling polytope^22^. An algorithm to alleviate the degeneracy problem was provided in^10^.

### Data availability

Everything necessary to reproduce the results we are reporting is included in the manuscript. Anyway, readers who want more information about data or protocols should contact the corresponding author.

## Electronic supplementary material

Supplementary Information
