# Supplementary material for: Maximally nonlocal Clauser-Horne-Shimony-Holt scenarios
Source: Sci Rep. 2018 May 8;8:7128. doi: 10.1038/s41598-018-24970-3 (PMC5940886; doi:10.1038/s41598-018-24970-3)
Supplement: Supplementary file 1 — Supplementary Information [file 41598_2018_24970_MOESM1_ESM.pdf]

Maximally nonlocal
Clauser-Horne-Shimony-Holt scenarios

Jesús Urías and José Manuel Méndez Martínez

Instituto de Física, UASLP
San Luis Potosí, SLP, México

1. Appendix A: Skeleton of the no-signaling polytope

The vertices of the no-signaling polytope produced by the enumeration algorithm in reference [10] are listed in Table A1. Horizontal lines in the table are organized in four segments. Each segment is a row of the 4×4 stochastic matrix of the box name given in the first column. All the data in Table A1 is parameterized by the following formulas

$$B_{\alpha a\,\beta b} = \begin{pmatrix} a+1 & a \\ \alpha+a+1 & \alpha+a \end{pmatrix} \otimes \begin{pmatrix} b+1 & b \\ \beta+b+1 & \beta+b \end{pmatrix} \quad \text{(mod 2)} \ ,$$

$$R_{ijk}(w_1 w_2 | x_1 x_2) = \begin{cases} 1/2, & w_1 + w_2 = (x_1 + i)(x_2 + j) + k \quad \text{(mod 2)} \\ 0, & \text{otherwise} \end{cases} \ .$$

The adjacency of PR boxes in the no-signaling polytope is given in Table A2. Horizontal lines correspond to PR boxes R_{ijk}, as indicated by the binary word ijk along the first column. The rest of columns in Table A2 correspond to B-boxes, as indicated by the binary word $\alpha a\,\beta b$ along the first two lines. A R-B edge of the no-signaling polytope is entered as a 1 in Table A2. Notice that the dual relationship between CHSH facets and PR boxes forbids the occurrence of edges between PR boxes in the no-signaling polytope.

	$x_1x_2 \rightarrow$	00	00	00	00	01	01	01	01	10	10	10	10	11	11	11	11
	$w_1w_2 \rightarrow$	00	01	10	11	00	01	10	11	00	01	10	11	00	01	10	11
*	B_{0000}	1	0	0	0	1	0	0	0	1	0	0	0	1	0	0	0
	B_{0001}	0	1	0	0	0	1	0	0	0	1	0	0	0	1	0	0
*	B_{0010}	1	0	0	0	0	1	0	0	1	0	0	0	0	1	0	0
	B_{0011}	0	1	0	0	1	0	0	0	0	1	0	0	1	0	0	0
	B_{0100}	0	0	1	0	0	0	1	0	0	0	1	0	0	0	1	0
*	B_{0101}	0	0	0	1	0	0	0	1	0	0	0	1	0	0	0	1
	B_{0110}	0	0	1	0	0	0	0	1	0	0	1	0	0	0	0	1
*	B_{0111}	0	0	0	1	0	0	1	0	0	0	0	1	0	0	1	0
*	B_{1000}	1	0	0	0	1	0	0	0	0	0	1	0	0	0	1	0
	B_{1001}	0	1	0	0	0	1	0	0	0	0	0	1	0	0	0	1
	B_{1010}	1	0	0	0	0	1	0	0	0	0	1	0	0	0	0	1
*	B_{1011}	0	1	0	0	1	0	0	0	0	0	0	1	0	0	1	0
	B_{1100}	0	0	1	0	0	0	1	0	1	0	0	0	1	0	0	0
*	B_{1101}	0	0	0	1	0	0	0	1	0	1	0	0	0	1	0	0
*	B_{1110}	0	0	1	0	0	0	0	1	1	0	0	0	0	1	0	0
	B_{1111}	0	0	0	1	0	0	1	0	0	1	0	0	1	0	0	0
*	R_{000}	$1/2$	0	0	$1/2$	$1/2$	0	0	$1/2$	$1/2$	0	0	$1/2$	0	$1/2$	$1/2$	0
	R_{001}	0	$1/2$	$1/2$	0	0	$1/2$	$1/2$	0	0	$1/2$	$1/2$	0	$1/2$	0	0	$1/2$
	R_{010}	$1/2$	0	0	$1/2$	$1/2$	0	0	$1/2$	0	$1/2$	$1/2$	0	$1/2$	0	0	$1/2$
	R_{011}	0	$1/2$	$1/2$	0	0	$1/2$	$1/2$	0	$1/2$	0	0	$1/2$	0	$1/2$	$1/2$	0
	R_{100}	$1/2$	0	0	$1/2$	0	$1/2$	$1/2$	0	$1/2$	0	0	$1/2$	$1/2$	0	0	$1/2$
	R_{101}	0	$1/2$	$1/2$	0	$1/2$	0	0	$1/2$	0	$1/2$	$1/2$	0	0	$1/2$	$1/2$	0
	R_{110}	0	$1/2$	$1/2$	0	$1/2$	0	0	$1/2$	$1/2$	0	0	$1/2$	$1/2$	0	0	$1/2$
	R_{111}	$1/2$	0	0	$1/2$	0	$1/2$	$1/2$	0	0	$1/2$	$1/2$	0	0	$1/2$	$1/2$	0

TABLE A1. Vertices of the no-signaling polytope.

$\underbrace{ijk}$	00	00	00	00	01	01	01	01	10	10	10	10	11	11	11	11	$\leftarrow \alpha a$
$\downarrow$	00	01	10	11	00	01	10	11	00	01	10	11	00	01	10	11	$\leftarrow \beta b$
000	1	0	1	0	0	1	0	1	1	0	0	1	0	1	1	0	
001	0	1	0	1	1	0	1	0	0	1	1	0	1	0	0	1	
010	1	0	0	1	0	1	1	0	1	0	1	0	0	1	0	1	
011	0	1	1	0	1	0	0	1	0	1	0	1	1	0	1	0	
100	1	0	1	0	0	1	0	1	0	1	1	0	1	0	0	1	
101	0	1	0	1	1	0	1	0	1	0	0	1	0	1	1	0	
110	1	0	0	1	0	1	1	0	0	1	0	1	1	0	1	0	
111	0	1	1	0	1	0	0	1	1	0	1	0	0	1	0	1	

TABLE A2. Adjacency matrix of the eight PR boxes in the no-signaling polytope.
